# Does gender matter? Exploring mental health recovery court legal and health outcomes

**DOI:** 10.1186/s40352-014-0012-0

**Published:** 2014-12-05

**Authors:** Catherine L Kothari, Robert Butkiewicz, Emily R Williams, Caron Jacobson, Diane S Morse, Catherine Cerulli

**Affiliations:** 1 Biomedical Sciences Department, Western Michigan University School of Medicine, Kalamazoo, 49008 MI USA; 2 Criminal Justice Services, Kalamazoo Community Mental Health and Substance Abuse Services, Kalamazoo, 49074 MI USA; 3 Criminal Justice, Department Governors State University, University Park, 60484 IL USA; 4 Department of Psychiatry, University of Rochester School of Medicine, Rochester, 14642 NY USA

**Keywords:** Mental health court, Gender and justice, Criminogenic factors, Healthcare utilization

## Abstract

**Background:**

Based upon therapeutic justice principles, mental health courts use legal leverage to improve access and compliance to treatment for defendants who are mentally ill. Justice-involved women have a higher prevalence of mental illness than men, and it plays a greater role in their criminal behavior. Despite this, studies examining whether women respond differently than men to mental health courts are lacking. Study goals were to examine gender-related differences in mental health court participation, and in criminal justice, psychiatric and health-related outcomes.

**Methods:**

This study utilized a quasi-experimental pre-posttest design without a control group. The data were abstracted from administrative records of Kalamazoo Community Mental Health and Substance Abuse agency, the county jail and both county hospitals, 2008 through 2011. Generalized estimating equation regression was used to assess gender-differences in pre-post program outcomes (jail days, psychiatric and medical hospitalization days, emergency department visits) for the 30 women and 63 men with a final mental health court disposition.

**Results:**

Program-eligible females were more likely than males to become enrolled in mental health court. Otherwise they were similar on all measured program-participation characteristics: treatment compliance, WRAP participation and graduation rate.

All participants showed significant reductions in emergency department visits, but women-completers had significantly steeper drops than males: from 6.7 emergency department visits to 1.3 for women, and from 4.1 to 2.4 for men. A similar gender pattern emerged with medical-hospitalization-days: from 2.2 medical hospital days down to 0.1 for women, and from 0.9 days up to 1.8 for men. While women had fewer psychiatric hospitalization days than men regardless of program involvement (2.5 and 4.6, respectively), both genders experienced fewer days after MHRC compared to before. Women and men showed equal gains from successful program completion in reduced jail days.

**Conclusions:**

Despite similar participation characteristics, findings point to greater health gains by female compared to male participants, and to lower overall psychiatric acuity. Mental-health-court participation was associated with decreased psychiatric hospitalization days and emergency department visits. Successful program completion correlated to fewer jail days for both women and men.

**Electronic supplementary material:**

The online version of this article (doi:10.1186/s40352-014-0012-0) contains supplementary material, which is available to authorized users.

## Background

### Therapeutic justice

The therapeutic justice movement grew from a recognition that crimes related to certain psychosocial problems such as addiction, domestic violence and mental illness were associated with higher recidivism and were less responsive to traditional criminal justice approaches (Winick [2002]). Within the therapeutic justice model, “problem solving” specialty courts are established that use legal leverage to address the root cause of criminal behavior (e.g., addiction, domestic violence or mental illness) through treatment and community resources (Hora et al. [1999]). Numerous studies have reported success implementing this model with drug courts, mental health courts, domestic violence courts, driving-while-intoxicated courts, and homicide courts (Wexler and Winick [1991]; Winick [1997]).

Studies focusing on the application of therapeutic jurisprudence using mental health courts have consistently documented improved psychiatric treatment and reduced criminal recidivism among participants after mental health court program completion, as compared to before, and as compared to treatment-as-usual control groups (Frailing [2010]; Goodale et al. [2013]; Herinckx et al. [2005]; Hiday and Ray [2010]; Steadman et al. [2011]). Recently, an additional approach, forensic assertive community treatment (FACT), has shown promise in preventing future crime among mentally ill individuals involved with the criminal justice system through assertive community outpatient treatment, which couples mental health treatment with comprehensive services including substance abuse, housing, transportation, and vocational counseling (Lamberti et al. [2004]).

Finally, despite the well-established link between criminal justice involvement and poor health (Arriola et al. [2006]; Belknap et al. [2012]; Choudhary et al. [2010]; Henneberger et al. [2014]; LaVene et al. [2003]; Woodson et al. [2010]), few studies have considered the potential health benefits that problem solving courts may offer (Frailing, [2010]; Steadman et al. [2011]). Benefits that are not just the result of keeping participants away from the adverse health conditions associated with incarceration, but also by improving conditions that mediate health (i.e., substance abuse, mental distress and violence).

### Women & therapeutic justice

The trend towards increased incarceration of women began in the 1980s, when, according to the National Center of Addiction and Substance Abuse, the rate of women going to prison increased 439 percent between 1980 and1995 (Olson [2000]). In more recent years, this trend has leveled off somewhat; from 1990 to 2009 women under correctional supervision increased from 14% to 18% of the population, with a complementary decrease in the proportion of men, from 86% to 82% (Glaze [2010]). The likelihood of a woman born in 2001 to be incarcerated at some point during her lifespan is 6 times higher than a woman who was born in 1974 (United States Department of Justice, Office of Justice Programs [2003]).

Compared to men, women involved in the criminal justice system are more likely to have accompanying psychosocial problems (mental illness, substance abuse problems, trauma histories), which play a greater role in their criminal justice involvement than in men’s (Covington and Bloom [2008]; DeHart [2008]; Green et al. [2005]; Grella et al. [2005]; James and Glaze 2006a; Lynch et al. [2012]; Steadman et al. [2009]). Studies conducted of female pretrial detainees found that more than 80 percent met criteria for one or more psychiatric disorders (Bloom and Covington [1998]) and approximately 22 percent were diagnosed with post-traumatic stress disorder (Vasey [1997]). Moreover, approximately eight out of ten female offenders diagnosed with a mental illness report histories of abuse (Bloom et al. [2004]). Co-occurring disorders (substance abuse combined with a mental health problem) among jailed women are significantly more prevalent than among jailed men (Bloom et al. [2004]). Additionally, criminal-justice–involved women are more likely than men to experience chronic medical problems, such as pulmonary and cardiovascular disease (Belknap et al. [2012]). Finally, justice-involved women tend to have greater family responsibilities than men, with 77% of surveyed female detainees reported providing most daily care for their children prior to incarceration (Austin and Irwin [2002]). Faced with a disproportionate share of family responsibilities, poorer health and a greater incidence of psychosocial stressors, women stand to benefit in unique, gender-specific ways from avoiding incarceration and being connected to the community resources available through problem-solving courts (James and Glaze [2006]).

In a test of women’s differential reaction to therapeutic justice programs targeting addiction, gender-responsive drug court programs were developed that offered treatment specifically addressing women’s unique trauma histories (James and Glaze [2006]; Orwin et al. [2001]; Webster et al. [2006]). A randomized control trial showed that women in a gender-responsive drug court treatment program stay enrolled longer and reported improved post-traumatic stress disorder symptomology compared to women in a mixed-gender drug court program, but were just as likely to have reduced substance use and decreased recidivism as mixed-gender participants (Mesina et al. [2012]). Despite these promising findings regarding psychiatric outcomes, no such examinations have been conducted for mental health court participants.

In sum, mental health courts, founded on the principles of therapeutic jurisprudence, use the arm of the law to increase access and compliance with treatment for mental health problems believed to underlie criminal behavior (Hora et al. [1999]; Winick [2002]). Given that mental illness is more tightly linked to criminal justice involvement among women compared to men (Covington and Bloom [2008]; DeHart [2008]; Green et al. [2005]; Grella et al. [2005]; James and Glaze [2006]; Lynch et al. [2012]; Steadman et al. [2009]), examination of gender differences in mental health problem-solving courts may yield insights into the relative importance of mental health treatment for mediating criminal involvement among women compared to men. Additionally, expanding the lens to include health-related outcomes in addition to psychiatric and criminal justice outcomes provides an opportunity to consider secondary effects of mental health court, including the degree to which they vary by gender.

#### Study purpose

This study provided a unique opportunity to examine the intersection of therapeutic justice, gender, crime, and health by reporting findings from a longitudinal study of 133 participants in a mental health court (30 women and 63 men). In particular, this study focused upon the role of gender on criminal justice and health-related outcomes associated with participation in mental health court. The study’s specific aims were:

##### Specific Aim #1 – gender differences in MHRC participation

To examine gender-related differences in program participation, including sanctions, length of participation and whether graduated or not.

##### Specific Aim#2 – gender differences in outcomes

To compare men and women’s pre- and post- program outcomes: number of days incarcerated, number of psychiatric hospitalization days and medical hospitalization days, and number of emergency department visits.

This article will report on the degree to which women responded differently to mental health court than men; an exploratory look given their relatively small group size.

## Methods

### Study design

This study utilized a quasi-experimental pre-posttest design without a control group (Trochim [2006]). Data were abstracted from archived records of Mental Health Recovery Court (MHRC) participants in Kalamazoo County Michigan during 2008 through 2011; all enrollees were included regardless of whether they successfully completed the program or not. Female and male participants were compared on pre-enrollment characteristics, their MHRC participation and the outcomes associated with MHRC, including their graduation or withdrawal from the program. The Kalamazoo Community Mental Health and Substance Abuse Agency Recipient Rights council provided permission to access data, and both Borgess Medical Center Institutional Review Board and Bronson Methodist Hospital Institutional Review Board provided institutional review board approval.

### Intervention: Kalamazoo Mental Health Recovery Court (MHRC) program

The primary purpose of MHRC is to divert adult offenders with serious mental illness and co-occurring (mental health, development disorder, substance abuse) disorders out of the traditional criminal justice track and into treatment. The Community Mental Health agency, Kalamazoo Community Mental Health and Substance Abuse Agency, administers the program, in collaboration with the Prosecuting Attorney’s Office, police agencies within Kalamazoo County, District Court and defense attorneys. Program components are: (1) Treatment and recovery services including WRAP (Wellness Recovery Action Plan), psychotropic medication as indicated, counseling, and case management as well as (2) intensive court supervision, with regular hearings attended by the mental health case manager and an MHRC peer specialist. WRAP, a peer-led group intervention focused upon self-efficacy and symptom monitoring (Copeland [2002]). Participation is strongly encouraged, but voluntary. MHRC is a phased program, with less supervision and fewer requirements as participants’ progress through the program. Participants may enter MHRC pre-conviction, as a diversion, or post-conviction, as a condition of probation. Program eligibility criteria are: Kalamazoo County residency, adult (age 18 or more), committed a misdemeanor-level crime and meets eligibility for case management services through Kalamazoo Community Mental Health and Substance Abuse Agency. After enrollment, participants may still fail to complete MHRC, either because they choose to withdraw or because their participation is terminated by the judge as a result of non-compliance with pretrial or probation bond conditions. Extensive efforts are made to prevent program failure, primarily through use of graduated sanctions such as increased testing for substance use, increased case management reporting, and brief jail stays (one to three days). Individuals who fail MHRC are then remanded to traditional court for conviction and/or sentencing.

### Study sample

The study sample consisted of all 133 individuals who were ever enrolled in Kalamazoo County MHRC from its inception in October, 2008 through the end of the study period in May, 2011. There was no exclusion criterion. Throughout the study period, 44 women and 89 men enrolled in MHRC.

The forty participants still actively enrolled in MHRC at the time of the study were excluded from the pre-post outcomes portion of the analysis. The remaining 93 individuals with final MHRC-dispositions had spent an average of 328 days in the MHRC program, ranging from a low of 30 days to a high of 699 days. The gender split of these 93 individuals was thirty women and sixty-three men.

As illustrated in Table 1, female and male enrollees were similar on many counts. They had similar demographic characteristics (age, race, employment and marital status), were equally likely to enter MHRC under diversion, and had similar criminal charges. For both women and men, roughly a quarter of the participants entered with assault-related charges (27.3% women and 28.1% men). . However, there were significant gender differences in psychiatric diagnoses at the time of MHRC entry, with the predominant diagnosis of bipolar disorder for women, and schizophrenia for men.

**Table 1:** **Pre-enrollment MHRC participant characteristics (N = 133)**

	MHRC participants		
	Women (44)	Men (89)	***p***value
**Demographics**			
Age mean (SD)	35.8 (12.0)	35.4 (13.5)	.853
Race % (#)			.125
White	61.4% (14)	59.6% (53)	
Black	31.8% (14)	34.8% (31)	
Hispanic	0.0% (0)	4.5% (4)	
Employment			.479
Not in labor force	25.0% (11)	29.2% (26)	
In labor force, unemployed	59.1% (26)	61.8% (55)	
In labor force, employed	15.9% (7)	9.0% (8)	
Marital status			.529
Not married	88.4% (39)	84.3% (75)	
Married	11.6% (5)	15.7% (14)	
**Charge Leading to MHRC % (#)**			.338
Domestic violence	20.5% (9)	15.7% (14)	
Assault, non-DV	6.8% (3)	12.4% (11)	
Substance use-related	27.3% (12)	14.6% (13)	
Ordinance violation	15.6% (7)	19.1% (17)	
Non-assault crime	29.5% (13)	38.2% (34)	
**Point of Entry into MHRC % (#)**			.348
Diversion	54.3% (19)	63.6% (49)	
Probation	45.7% (16)	36.4% (28)	
**Principle Psychiatric Diagnosis At MHRC Enrollment % (#)**			.034
Schizophrenia	13.6% (6)	36.0% (32)	
Mood disorder	22.7% (10)	24.7% (10)	
Bipolar disorder	38.6% (17)	22.5% (20)	
Psychotic disorder	4.5% (4)	5.6% (5)	
Borderline disorder	13.6% (6)	2.2% (2)	

NOTE: Percentages may not add to 100% due to the removal of categories with gender sub-groups that have only a single member.

### Setting

The judicial system in the study county has long embraced specialty courts, beginning in 1992, with Women’s Drug Treatment Court. At the time of the study, MHRC was one of seven specialty courts, which included four drug courts (women, men, juvenile and family), a Sobriety Court (for drunk driving offenders) and a Domestic Violence Court. Compared to male Kalamazoo Community Mental Health and Substance Abuse Agency consumers, female consumers were disproportionately more likely to be booked into jail relative to their proportion in the general county population, with relative ratios of 5.6 for women and 2.5 for males, respectively (Kothari and Butkiewicz [2013]).

### Data collection and measures

Study data were generated through secondary analysis of administrative records from the MHRC program, Kalamazoo Community Mental Health and Substance Abuse Agency, Kalamazoo County Sheriff’s Department, and two local hospitals, Borgess Medical Center and Bronson Methodist Hospital. Data was collected for each participant for a period spanning one year prior to MHRC enrollment through the date of data collection in May, 2011.

Medical record data were collected in two stages: (1) Manual abstraction of Borgess Medical Center and Bronson Methodist Hospital medical record numbers and (2) submission of the set of medical record numbers to each hospital for extraction by the hospital Health Information Management Departments into visit-level datasets. Jail data collection also occurred in two stages: (1) Extraction of the total population of jail stays into a dataset by the Kalamazoo County Sheriff’s Information Technology Department, and (2) Electronic data linkage to MHRC participants using Link Plus 2.0, an algorithm-based matching software developed by the CDC. Linkage was based upon first and last names and date of birth, as noted in MHRC records. Psychiatric hospitalization data were obtained from Kalamazoo Community Mental Health and Substance Abuse Agency records, and from Borgess Medical Center data, which has a psychiatric in-patient unit.

### MHRC descriptors

Demographic and program participation characteristics were abstracted from MHRC program records. Demographic variables included the gender variable as well as age, race, employment and marital status. Program participation variables included the criminal charge leading to MHRC entry, the principle psychiatric diagnosis at entry, whether MHRC participation was through diversion or probation, completion of WRAP, medication and substance abuse treatment compliance, and whether MHRC-related jail sanctions were imposed. Additionally, MHRC program disposition (whether graduated or failed the program) was abstracted.

### Pre-post outcomes

Four outcome measures, serving as proxies for criminal justice involvement and health, were tracked for the year prior to program enrollment (pre) and the period after leaving the program (post): Jail bookings, psychiatric hospitalization, medical hospitalization, and emergency department visits. Jail bookings may have been the result of a variety of situations: New arrest (followed by either release or prosecution), post-conviction sentencing, or violations of probation, pretrial bond or restraining order. Prevalence and magnitude was calculated for each measure. Prevalence was the percent of individuals experiencing a particular outcome during the period under study. Magnitude was operationalized as the total number of days spent in jail or the hospital (psychiatric or medical), and, for the emergency department, the total number of visits during the study period. Days were computed based upon admission and discharge dates, and calculations included the actual day of admission. Because participants had rolling MHRC-enrollment dates and different lengths of program participation, their “post” periods varied, from 24 days to 902 days, with a mean of 377 days. To facilitate post-period comparisons, annualized rates were computed for the magnitude measures using the following equation: Rate = [(# days or visits) / (# days in “after” period)] x 365.

### Analysis

#### Specific Aim #1 – gender differences in MHRC participation

Bivariate statistical comparisons between female and male MHRC participants for categorical variables were conducted using Pearson’s Chi-Square. Fisher’s Exact Test was used when cell counts dropped below five. Bivariate comparisons of continuous variables were conducted using one-way ANOVA.

#### Specific Aim#2 – gender differences in outcomes

Generalized estimating equation regression (GEE) was used to estimate the association of gender with each outcome (jail day rate, psychiatric day rate, emergency department visit, medical hospitalization day rate). Mixed-modelling with GEE was conducted, where the repeated measures were pre-post MHRC outcomes and the fixed measures were gender and program-completion. Main effects were calculated for gender, pre-post MHRC period, and program completion. Two-way interaction effects were estimated for gender-with-MHRC and for gender-with -completion by creating a four-level variable for each interaction (e.g., 1. female/pre-MHRC, 2. female/post-MHRC, 3. male/pre-MHRC, 4. male/post-MHRC) and entering them into the regression model. Unstandardized beta coefficients and their associated confidence intervals were reported, and served as adjusted effect sizes for each factor (Breaugh [2002]; Grissom and Kim [2012]). Given the moderate sample sizes, only the primary variables of interest described above were included in the regression models; no additional covariates were included. All tests were conducted with 2-tailed significance and the significance level set at *p* < .05. Data analyses were completed using SPSS version 20.0.

## Results

### Specific Aim #1 – gender differences in MHRC participation

Eligible women were more likely to be enrolled in MHRC than eligible men. Of the 237 female Kalamazoo Community Mental Health and Substance Abuse Services consumers booked into jail in a single year, 2009, 8.9% (21) were enrolled in MHRC compared to 3.4% MHRC participation among male consumers booked into jail the same year (18 of 537). As illustrated in Table 2 below, there were no gender-related differences regarding MHRC participation Women and men completed the WRAP program at the same rates, had similar medication compliance, similar rates of jail sanctions imposed, and similar rates of substance abuse treatment and treatment compliance noted.

**Table 2:** **Gender comparison of MHRC-participation characteristics (N = 133)**

	MHRC participants		
	Women (44)	Men (89)	***p***value
Completion of WRAP Program*	59.5% (25)	62.9% (56)	.709
Medication Compliance (as indicated)**	100% (38)	98.8% (83)	.499
MHRC jail sanctions	27.3% (12)	31.5% (28)	.625
Substance Abuse Noted	68.3% (30)	64.0% (57)	.637
(Among those with Subs. Abuse Notations)	(30)	(57)	
Substance Abuse Progress	60.0% (18)	56.1% (32)	.729
MHRC Disposition			.455
Completed MHRC	38.6% (17)	38.2% (34)	
Still Participating in MHRC	31.8% (14)	24.7% (22)	
Failed MHRC (Terminated or Withdrew)	29.5% (13)	32.6% (29)	
Administrative Discharge	0	4.5% (4)	

*Wellness Recovery Action Plan **Percentages based upon non-missing.

Most significantly, women and men successfully completed the MHRC program at the same rates. At the time of the study, 40 participants were still active participants in the program and 93 had had a final MHRC disposition (either completion or failure). Of the sixty individuals failing the program, fifty-six been removed from the program for non-compliance four had been removed at their own request. Of the 93 with a final MHRC disposition, 30 were women and 63 were men. Of these, 17 women (56.7%) and 34 men (54.0%) successfully completed MHRC. There were no differences in length of MHRC participation by gender: women had an average length of 313 days and men had an average length of 328 days (*p* = .655).

### Specific Aim#2 – gender differences in outcomes: rates prior to MHRC-participation

As demonstrated in Table 3, women and men also did not vary significantly regarding their criminal or health histories in the year prior to MHRC enrollment. Across gender, 37.6% of MHRC participants had a psychiatric hospitalization in the year prior to MHRC (total figures reported in text, not shown). Those hospitalized spent an average of one week in the hospital. Emergency department utilization was quite high across the board, with 84.9% visiting the emergency department the year before MHRC, for an average of seven visits. One out of five (20.4%) participants had a medical hospitalization prior to enrollment; those that did stayed for an average of 6.6 days. Consistent with the criminal justice involvement that was the basis for their MHRC participation, a majority (80.6%) had one or more jail bookings in the former year, *p* = .073.

**Table 3:** **Rates in the Year Prior to MHRC Participation, Stratified by Gender (N = 93)**

	Pre-MHRC		
	Women (30)	Men (63)	***p***
**Criminal Justice**			
Mean # of Days in Jail (CI):	24.0 (6.7, 41.4)	11.3 (5.9, 16.6)	.073
- % with Jail Booking	83.3% (25)	79.4% (50)	.651
-Mean days among those w/ booking	28.8 (8.2, 49.4)	14.2 (7.7, 20.7)	.086
**Psychiatric Hospitalization**			
Mean # of Days in Hospital (CI):	2.3 (0.6, 4.0)	3.6 (2.2, 5.0)	.256
-% with Any Psych. Hospitalization	26.7% (8)	42.9% (27)	.132
-Mean days among hospitalized	7.2 (1.5, 12.9)	7.4 (5.0, 9.8)	.928
**Emergency Department Visits**			
Mean # of Visits (CI):	5.5 (2.1, 9.0)	6.4 (3.9, 8.9)	.674
- % with Any Visit	86.7% (26)	84.1% (53)	.749
-Mean visits among those w/ any	6.4 (2.5, 10.3)	7.7 (4.8, 10.5)	.602
**Medical Hospitalization**			
Mean # of Days in Hospital (CI):	2.3 (0.2, 4.7)	0.9 (0.2, 1.7)	.173
- % with Any Medical Hosp.	26.7% (8)	17.5% (11)	.303
-Mean days among those hosp.	8.5 (−0.5, 17.4)	5.2 (1.6, 8.9)	.400

### Specific Aim#2 – Gender differences in outcomes: mixed-modelling with GEE

The results of the multivariate regression, taking into account whether MHRC was successfully completed or not, more clearly reveal the differential association of gender with MHRC outcomes, an association that varied depending upon the outcome (Table 4).

**Table 4:** **Gender differences in outcomes: mixed-modelling with GEE (N = 93)**

			Annual rate		Confidence interval	***p***
			Mean, CI	β*		
JAIL	MHRC	Pre	15.4 (8.8, 22.0)	(ref)	--	
		Post	15.7 (7.9, 23.4)	5.1	(−5.3, 15.5)	.335
	Gender	Male	13.8 (8.1, 19.6)	(ref)	---	
		Female	19.1 (9.0, 29.1)	15.1	(−8.6, 38.8)	.211
	Completion	Failed	25.4 (15.6, 35.1)	(ref)	---	
		Completed	7.4 (3.4, 11.4)	−17.0	(−29.3, −4.8)	.006
	Gender X MHRC		---	−15.0	(−36.9, 6.9)	.180
	Gender X Completion		---	−3.4	(−26.3, 19.6)	.773
PSYCH	MHRC	Pre	6.0 (3.6, 8.4)	(ref)	--	
		Post	1.8 (−0.02, 3.6)	−4.8	(−8.5, −1.1)	.011
	Gender	Male	4.6 (2.5, 6.7)	(ref)	---	
		Female	2.5 (0.5, 4.4)	−5.8	(−9.9, −1.7)	.006
	Completion	Failed	3.6 (1.3, 5.9)	(ref)	---	
		Completed	4.1 (2.1, 6.2)	−1.0	(−5.3, 3.4)	.663
	Gender X MHRC		---	1.8	(−3.2, 6.8)	.475
	Gender X Completion		---	4.9	(−6.2, 10.4)	.082
ED	MHRC	Pre	6.2 (4.2, 8.2)	(ref)	--	
		Post	3.6 (2.1, 5.0)	−2.1	(−4.1, −0.2)	.032
	Gender	Male	5.4 (3.8, 7.0)	(ref)	---	
		Female	3.8 (1.9, 5.6)	−3.9	(−8.7, 1.0)	.118
	Completion	Failed	6.5 (4.2, 8.8)	(ref)	---	
		Completed	3.5 (2.3, 4.7)	−4.7	(−8.7, −0.6)	.024
	Gender X MHRC		---	−1.4	(−5.5, 2.7)	.505
	Gender X Completion		---	5.4	(0.3, 10.5)	.038
MED INPT	MHRC	Pre	1.3 (0.4, 2.2)	(ref)	--	
		Post	1.3 (0.4, 2.2)	0.9	(−0.1, 1.9)	.075
	Gender	Male	1.4 (0.6, 2.1)	(ref)	---	
		Female	1.2 (−0.01, 2.4)	1.4	(−2.3, 5.1)	.452
	Completion	Failed	1.6 (0.5, 2.8)	(ref)	---	
		Completed	1.0 (0.4, 1.7)	−0.6	(−2.4, 1.3)	.566
	Gender X MHRC		---	−3.0	(−5.5, −0.5)	.018
	Gender X Completion		---	−0.1	(−3.3, 3.0)	.929

*Unstandardized beta coefficient representing the effect of each predictor upon days or visits, depending upon the outcome variable.

#### Jail

The only factor predicting number of jail days was whether or not MHRC was successfully completed. As indicated by the β coefficient in Table 4, participants successfully completing MHRC had a mean annualized rate of seventeen fewer jail days in the post period than participants who withdrew or were terminated prematurely from MHRC.

#### Psychiatric hospitalization

In contrast, the outcome psychiatric hospitalization days showed significant main effects for both MHRC, as indicated by the “MHRC pre-post” factor, and gender; with a rate of nearly five fewer psychiatric hospitalization days after MHRC participation compared to before (β coefficient of −4.8), and a rate of nearly six fewer days for women compared to men as indicated by a β coefficient of −5.8 days among women. By taking into account MHRC participation and examining psychiatric hospitalization across the entire study period, this multivariate analysis revealed the gender effect of women’s lower psychiatric hospitalizations, a finding that was not apparent in the bivariate, pre-MHRC results reported in Table 3.

#### Emergency department visits

Similar to psychiatric outcomes, there was a significant main effect for MHRC, with a β coefficient of −2.1 visits after MHRC compared to before. The degree to which program completion led to changes in emergency department visits, however, varied by gender: women completers had the highest pre-MHRC levels and showed the biggest drops, from an annualized average of 6.7 emergency department visits to 1.3. Women failing MHRC went from 3.8 visits to 2.9. In contrast, men who failed had the highest emergency department visits both pre- and post-MHRC, although even they experienced improvement, going from 9.2 annualized average number of visits to 6.6. Men who completed MHRC went from 4.1 to 2.4.

Medical Hospitalization: Medical hospitalization days also varied by gender: prior to MHRC, women had more medical hospitalization days, but these dropped precipitously in the post period, from an annualized average of 2.2 days to 0.1 days for women. Men showed the opposite trend: going from an annualized average of 0.9 days before MHRC to 1.8 days after.

## Discussion

Among a study population that was demographically similar to mental health court participants across the nation (Steadman and Naples [2005]), study findings revealed important gender-related patterns in participation as well as in criminal justice, psychiatric and health-related outcomes.

### Specific Aim #1 – gender differences in MHRC participation

Consistent with previous research among jailed consumers (Steadman and Naples [2005]), women were more likely to be MHRC participants than men. Luskin ([2001]) found that mental health court referral decisions tended to favor females, perceiving them as less risky to release into the community, regardless of their presenting criminal charge. Otherwise, their engagement with MHRC was very similar to men’s. There were no differences regarding men and women’s portals of entry into the MHRC, suggesting that screening for mental health for both women and men might be implemented in probation and diversion settings. They were just as likely to complete WRAP, to comply with program requirements (as indicated by medication and substance use compliance and jail sanctions), and to successfully complete the MHRC program. While this is the first study to explicitly examine gender-related differences among mental health court participants, drug court studies have found higher completion rates among women (Gray,’05); a finding that has been attributed to women’s greater motivation for both mental health and substance abuse treatment within drug court settings (Webster et al. [2006]).

Contrary to trends among the general criminal justice population, there were few sociodemographic differences by gender (Covington and Bloom [2008]; DeHart [2008]; Green et al. [2005]; Grella et al. [2005]; James and Glaze [2006]; Lynch et al. [2012]; Steadman et al. [2009]). Study women entering MHRC were similar to men on age, race, employment, marital status and criminal charges. The prevalence of substance-related criminal charges by women were lower than that documented in other studies, perhaps due to the co-existence of a women’s drug court in the study community (Bloom et al. [2004]; Chesney-Lind [2002]; Morse et al. [2013]) The psychiatric diagnostic difference found between women and men supports earlier work showing that among community samples, more men suffer from schizophrenia than women (Piccinelli and Homen [1997]). It is possible that comorbid mental illness and justice involvement become equalizing factors, suppressing traditional gender differences.

### Specific Aim#2 – gender differences in health outcomes

Study findings suggest that, despite similar baseline levels, female participants may have differential health-related responses to MHRC than males, as indicated by the interaction effects of gender with MHRC-completion for emergency department visits, and gender with pre-post MHRC for medical hospitalizations. Although all participants demonstrated reductions in emergency department visits after MHRC compared to before, females who completed the program had the most dramatic drops, compared to males who had completed the program and compared to those of both genders who failed MHRC. While just as high as men’s in the year before MHRC enrollment, women had steeper drops than males in emergency department utilization and inpatient medical-hospitalization-days after program participation compared to before.

A similar pattern was observed for medical hospitalization days, with women having more hospitalization days than men prior to MHRC and less days than men after MHRC, regardless whether they had completed or had failed MHRC. Although one of the first studies to document reduced acute medical utilization by mental health court participants, these outcomes are consistent with prior findings that both mental health and drug court participation are associated with reduced homelessness, improved daily functioning, reduced substance use and improved mental health symptoms, factors that affect emergency department utilization as well as healthy living practices (Hunt et al. [2006]; Remington et al. [2010]; Steadman and Naples, [2005]; Tyuse and Linhorst [2005]; Webster et al. [2006]). These reductions are particularly notable, given that women in the general population tend to have higher emergency department visits as well as medical hospitalizations than males (Centers for Disease Control and Prevention (CDC), National Center for Health Statistics, 2013; National Center for Health Statistics and Prevention [2013]), and that criminal-justice-involved women have higher rates of chronic disease than their male counterparts (Belknap et al. [2012]). For women, improved integrated care through MHRC may help them address these multiple complex areas of their lives, and result in their needing fewer acute healthcare services.

### Specific Aim#2 – gender differences in psychiatric outcomes

Gender was also found to be significantly related to psychiatric hospitalization days, with women having fewer psychiatric hospitalization days compared to men. Women had fewer psychiatric hospitalizations than men whether they completed MHRC or not. This may be linked to lower mental health acuity among MHRC-eligible women compared to MHRC-eligible men, to greater psychiatric treatment adherence by women regardless of MHRC-participation or to the higher rates of schizophrenia among male MHRC participants; a condition that is generally associated with greater psychiatric hospitalizations (Blader and Carlson [2007]; Klinkenberg & Calsyn, [1998]). This suggests that, while more criminal-justice-involved women than men may have mental illness, mental health court evokes the same response among program-eligible women as among program-eligible men.

As has been found in other mental health court studies, MHRC participation by itself is strongly and significantly linked to reduced psychiatric hospitalization days, across genders and across program discharge status (e.g., whether completed MHRC or not) (Frailing [2010]; Goodale et al. [2013]; Herinckx et al. [2005]; Hiday and Ray [2010]; Steadman et al. [2011]). This speaks to the effectiveness of MHRC in facilitating mental health treatment within an accountability structure that supports treatment compliance, with equal gains experienced by women and men.

Considering that a psychiatric diagnosis was one of the few characteristics on which women and men varied, it may be that the nature of women’s psychiatric illness is a factor in a heightened health-related response to MHRC. Bipolar diagnoses have been associated with somatoform disorders (experiencing psychological distress in the form of physical maladies) and can be associated with iatrogenic harm (Kroenke [2007]; Morse et al. [1997]; Smith et al. [2009]). Hence improved psychiatric outcomes in somatization could translate into fewer days of needed medical treatment with fewer ensuing complications. Somatization tends to be more common among women compared to men in general, especially women with drug and trauma histories (Lieb et al. [2002]; Waitzkin and Magana [1997]); histories that are well-documented among justice-involved women (Covington and Bloom [2008]; DeHart, [2008]; Green et al., [2005]; Grella et al., [2005]; James and Glaze 2006a; Lynch et al., [2012]; Steadman et al., [2009]). Reducing this distress, through mental health treatment may have greater health benefit for women.

### Specific Aim#2 – No gender differences in jail outcomes

Among study outcomes, gender appears to have the least relevance for jail outcomes. The multivariate analysis revealed that the most important predictor of days in jail was successful completion of MHRC, regardless of gender. Those that graduated from the program had a mean of 7.4 days in jail, compared to those who failed the program and have a mean of 25.4 days. Previous studies documenting lower recidivism by mental health court women compared to men have also reported higher program completion rates among women (Frailing, [2010]; Center Gains [2010]); current study results point to program completion, rather than gender, as the operating factor. Aside from program completion, prior number of arrests and ongoing substance abuse have been consistent predictors of criminality within the general population as well as mental health court participants (Case et al. [2009]; Center Gains [2010]; Gendreau et al. [1996]; Steadman et al. [2011]); characteristics that were equally high among study women as among study men.

### Limitations

As with all studies, there are several limitations worth noting. This study was conducted in one setting, which limits generalizability. Further, this community had a well-coordinated, community-based mental health treatment option for court-involved defendants. In other mental health courts where care is not as readily accessible, findings may vary. Another limitation is that the study had no direct measures of health or criminality, instead relying upon proxy measures (jail, hospitalization, emergency department visits) that may incorporate system-related biases that undercounted or overcounted program effects. The small sample size may have limited the power of the study to detect true differences in outcomes between men and women. Lastly, this data set is unable to demonstrate a causal relationship due to the study design. However, this unique data set uses multivariate analysis of direct and interaction effects that uncovered key gender-related relationships, and combined data from multiple sources to examine a complex question from multiple perspectives: how do we help justice involved men and women with mental health disorders to rebuild their lives?

This study adds to the literature by documenting the differential health gains by female mental health court participants; gains that were not tied to measurable differences between women and men’s changes in psychiatric hospitalization or jail bookings. Just as importantly, MHRC participation, independent of gender, was associated with reduced jail days overall. This effect was restricted to those who completed the program; unlike psychiatric outcome improvements that were seen for all participants regardless of completion (Frailing, [2010]; Goodale et al. [2013]; Herinckx et al. [2005]; Hiday and Ray [2010]; Steadman et al. [2011]). That MHRC is therapeutic for both genders suggests that policy makers may want to reconsider traditional outcome measures of recovery court participant’s “success” or “treatment completion”. If linking with a MHRC, regardless of graduation results in improved appropriate health care and less urgent care use (inpatient psychiatry and emergency department), it stands to reason that this effect could be a viable cost saving measure for local communities

## Conclusions

Although similar to male participants in several regards, the greater health benefit experienced by women MHRC participants provides preliminary support for the differential impact of therapeutic justice approaches upon women defendants; a finding that warrants further investigation. Unlike the general criminal justice population, female and male mental health court participants had similar demographics, criminal histories, substance use and program-participation characteristics. Notably, women presented with different psychiatric diagnoses and lower acuity. Despite this, both genders responded to mental health court with reduced psychiatric hospitalization days and reduced jail days. Importantly, women showed differential health gains, with steeper drops than men in emergency department visits and medical hospitalization days.

## Authors’ original submitted files for images

Below are the links to the authors’ original submitted files for images. Authors’ original file for figure 1

## Abbreviations

MHRC: Mental health recovery court
WRAP: Wellness recovery action plan
GEE: Generalized estimating equation
DV: Domestic violence
ED: Emergency department
