# Supplementary figures and images for: Does gender matter? Exploring mental health recovery court legal and health outcomes
Source: Health Justice. 2014 Dec 5;2:12. doi: 10.1186/s40352-014-0012-0 (PMC4269165; doi:10.1186/s40352-014-0012-0)

Figure 1. Number of Women who are CMH Consumers and Booked into Jail, 2009

| 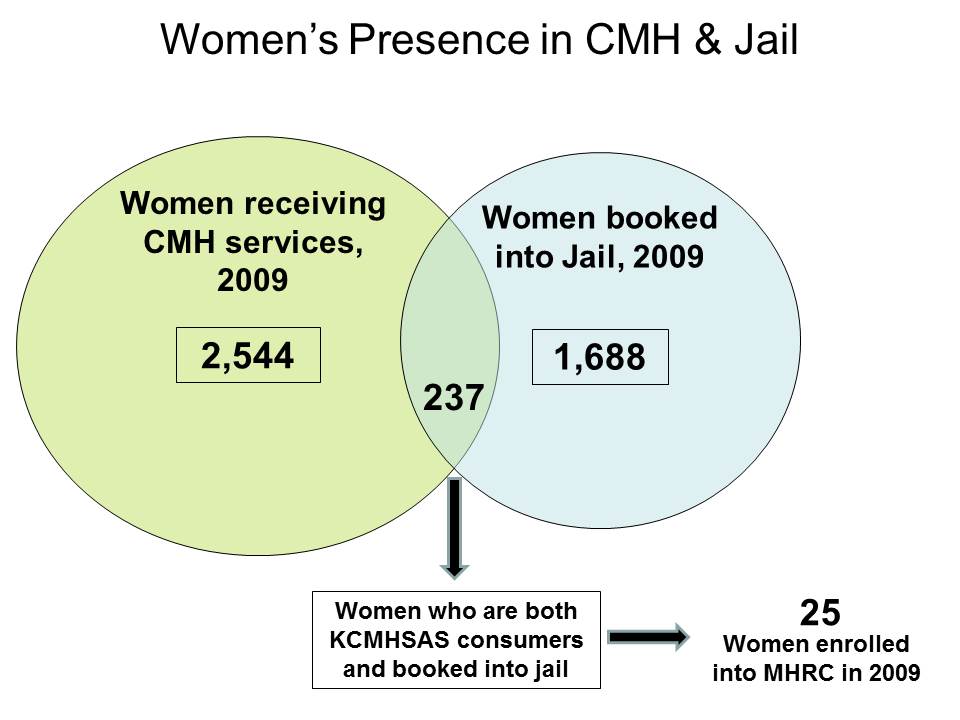 |
| --- |

Supplement: Supplementary file 1 — Authors’ original file for figure 1 [file 40352_2014_12_MOESM1_ESM.docx]
